# Successful Endoscopic Management of Suspected Foreign Bodies in Upper Gastrointestinal Tract among Patients Undergoing Upper Gastrointestinal Endoscopy in a Tertiary Care Hospital: A Descriptive Cross-sectional Study

**DOI:** 10.31729/jnma.6714

**Published:** 2021-07-31

**Authors:** Ramila Shrestha, Shankar Baral, Mukesh Sharma, Jiwan Thapa, Dibas Khadka

**Affiliations:** 1 Department of Gastroenterology, National Academy of Medical Sciences, Bir Hospital, Kathmandu, Nepal

**Keywords:** *endoscopes*, *foreign bodies*, *gastrointestinal tract*

## Abstract

**Introduction:**

Most ingested foreign bodies pass through the gastrointestinal tract spontaneously. However, some foreign bodies may get impacted in the upper gastrointestinal tract. A variety of endoscopic techniques and instruments are indicated for the removal of such impacted foreign bodies. This study was conducted to find out the prevalence of successful endoscopic removal of foreign bodies.

**Methods:**

This descriptive cross-sectional study was conducted among patients who presented at the Department of Gastroenterology with complaints of upper gastrointestinal foreign body ingestion from 2/11/2008 to 23/07/2020 after taking ethical approval of the research proposal was taken from Institutional Review Board (Reference no 13). Convenient sampling was done. The data were entered into Microsoft Excel and analyzed in Statistical Package of Social Sciences version 22.

**Results:**

A total of 119 cases were identified with foreign bodies ingestion. In hundred patients, foreign bodies 100 (84 %) (77.41-90.58 at 95% Confidence Interval) were extracted completely. Complete extraction failed in 19 (16%) patients. Six (5%) patients were treated by push technique and 10 (8.4%) patients with failed retrieval, received surgical intervention for foreign body removal.

**Conclusions:**

Endoscopic removal technique of foreign bodies in the upper gastrointestinal tract was successful in most of the cases and is associated with few complications.

## INTRODUCTION

The impaction of an ingested substance is a frequent cause of gastrointestinal emergencies.^1^ About IQ-20 percent of such ingested foreign bodies require an endoscopic removal and less than one percent of patients will require surgical removal.^2^ In adults, impaction of bone or meat bolus occurs during eating.^3^ Impaction of non-food objects occurs in patients with psychiatric disorders, developmental delay, and alcohol intoxication.^4^ Foreign bodies often lodge in the esophagus because of the object size, shape, narrowing of the esophagus lumen, or anatomic abnormalities.^5-7^

Patients with esophageal foreign bodies are almost always symptomatic.^8-9^ Potential complications of esophageal foreign bodies include laceration, punctures with associated abscess, perforations, mediastinitis, pneumomediastinum, pneumothorax, and injuries to the aorta or pulmonary vasculature.^10^ Various endoscopic techniques and instruments are used for the removal of these foreign bodies.^7,11^

Our study aimed to find out the prevalence of successful endoscopic removal of foreign bodies.

## METHODS

A descriptive cross-sectional study was conducted after obtaining ethical approval from the Institutional Review Committee (Reference number No.13) of the National Academy of Medical Sciences. Data was collected from 20/01/2008 to 23/07/2020 retrospectively. The study was conducted at Gastroenterology Unit, Medicine Department, National Academy of Medical Sciences, Bir Hospital. The study included all patients with a history of impacted foreign bodies, confirmed by endoscopy in more than 18 years of age group. We excluded patients with a history of ingested foreign bodies but not detected on endoscopy, patients referred to other centers, incomplete records, or who underwent direct surgical intervention. All medical records including age, sex, type of foreign body, associated signs and symptoms, used endoscopic devices were reviewed. The sample size was calculated as follows,

n = Z^2^ × p × q / e^2^

  = (1.96^2^) × (0.956) × (1-0.956) / (0.04)^2^

  ≈ 101

Where,

n = required sample size
Z = 1.96 at 95% Confidence Interval (CI)
p = prevalence of success rate in foreign body extraction as per Emra, et al. 95.6%^16^
q = 1-p
e = margin of error, 4%

Taking the non-response rate 10%, the calculated sample size is 111.

However, 119 participants were included in our study. Convenient sampling was done. Written consent was obtained from all the participants.

Various instruments (Forceps and Dormia baskets) were used to retrieve the impacted foreign bodies. Repeat endoscopy was done after the foreign body retrieval.

The data were entered into Microsoft Excel and analyzed in Statistical Package of Social Sciences version 22.

## RESULTS

The total number of participants in our study was 119. In 100 (84%) (77.41-90.58 at 95% Confidence Interval) patients, foreign bodies were extracted completely. Complete extraction failed in 19 (16%) patients. Among patients in whom the foreign body was extracted completely, 58 (79.5%) were male patients and 42 (91.3%) were female patients. Six (5%) patients were treated by push technique and 10 (8.4%) patients with failed retrieval, received surgical intervention for foreign body removal.

The percentage of complete removal was only 5 (56%) for <25 years of age but nearly 34 (87%) for 25-49 years and 40 (87%) in 50-74 years. Complete removal was 21 (83.3%) for the 75 years and above age group. About 42 (92%) of females and 58 (79.5%) of male patients had complete removal of foreign body. About 89 (87%) of cases with food as the foreign body had complete removal whereas 11 (68.8%) of non-food items were completely removed. Around 89 (83%) of the items located in the esophagus were removed completely whereas 11 (100%) of the non-esophagus items were removed. The complete removal percentage using forceps and Dormia basket was nearly the same (Table 1).

**Table 1:** Complete and failed removal in different groups.

Age	Complete removal n (%)	Failure to remove completely n (%)	Total n (%)
<25	5 (55.6)	4 (44.4)	9 (7.5)
25-49	34 (87.2)	5 (12.8)	39 (32.77)
50-74	40 (87)	6 (13)	46 (38.65)
75 +	21(84)	4 (16)	25 (21.00)
**Sex**			
Female	42 (91.3)	4 (8.7)	46 (38.65)
Male	58 (79.5)	15 (20.5)	73 (61.34)
**Type of Foreign body**			
Food	89 (86.4)	14 (13.6)	103 (86.55)
Non-food	11 (68.8)	5 (31.2)	16 (13.44)
**Foreign body location**			
Esophagus	89 (82.4)	19 (17.6)	108 (90.75)
Non Esophagus	11 (100)	0	11 (9.2)
**Accessories**			
Forceps	42 (84)	8 (16)	50 (42.01)
Dormia Basket	58 (84.1)	11 (15.9)	69 (57.98)

Seventy-three (61.3%) patients were male and 46 (38.3%) were female. One hundred and three (86.6%) cases of ingested foreign bodies were food whereas nonfood were in 16 (13.4%) patients respectively. Chicken bone and chicken were found in 51 (42.9%) patients and 29 (24.4%) patients respectively. Some patients had ingested fruit seeds, lapsi in 7 (5.9%) patients, and mango seed in 1 (0.8%). Other ingested meat like buff and mutton were observed in 6 (5%) patients and 4 (3.4%) patients respectively. Fishbone was found in 3 (2.5%). Jack fruit, a lump of food, vegetables were visualized in 1 (0.8%). True foreign bodies (nonfood) were found in 16 (13.4%). Five (4.2%) had accidentally ingested dentures. The wire was seen in 4 (3.3%) patients with denture impaction. Coin, hair, part of Percutaneous Endoscopic Gastrostomy (PEG) tube, pin, stone, and two buttons were observed in only 1 (0.8%) (Table 2).

**Table 2:** Profile of patients with upper gastrointestinal tract foreign bodies (n = 119).

Age (years)	n (%)
<25	9 (7.6)
25-49	39 (32.8)
50-74	46 (38.7)
75 +	25 (21.0)
**Sex**	
Female	46 (38.7)
Male	73 (61.3)
**Type of foreign body**	
Food	103 (86.6)
Non-food	16 (13.4)
**Foreign body location**	
Esophagus	108 (90.8)
Non-esophagus	11 (9.2)
**Accessories**	
Forceps	50 (42.0)
Dormia basket	69 (58.0)
**Outcome**	
Complete removal	100 (84.0)
Failure to remove completely	19 (16.0)

About 46 (39%) of cases were of age 50-74 followed by 25-49 of 39 (32.8%), 75 and above the age 25 (21%). About 9 (8%) of cases were less than 25 years of age. Nearly 103 87% of the cases were related to food items. In about 108 (91%) of the location was esophagus. The most widely used tool was the Dormia basket followed by Shark tooth forceps, Crocodile forceps, and rat tooth forceps. 69 (58%) of cases were treated with a Dormia basket and the remaining using forceps.

## DISCUSSION

In this study, a total number of 11 9 patients had foreign body impaction in the upper gastrointestinal tract. Endoscopic procedures were performed in almost all patients within 24 hours of attending the hospital. Foreign body impaction was more prevalent in male patients (61.3%). These findings were consistent with several studies from eastern and western regions.^12-17^. Our study concluded that the most common foreign bodies were food boluses, located in the esophagus (90.8%). Various other studies have suggested the esophagus as the common site of impaction of foreign bodies.^12,13,16,18-20^ Mosca S, et al. showed almost all the foreign bodies were found in the esophagus.^21^

In a prospective study done by Shrestha D, et al. from Nepal in patients with a clinical diagnosis of esophageal foreign body from July 2013 to July 2017, out of 1 01 cases of esophageal foreign body, there were 57% cases of chicken bone, followed by 17% mutton bone and 12% meat bolus which was consistent with our study.^23^ A study from Manipal Teaching Hospital by Koirala K, et al. compared esophageal foreign bodies in pediatric and adult populations the result of which showed that out of total cases of foreign body impaction, 44.4% of patients were children and 55.5% were adults. The coin was the most common foreign body in the pediatric population (82.1%) whereas bones were the commonest ones (91.4%) in adults.^24^ Two previous studies from Nepal showed chicken bones were a common foreign body.^25-6^ Bony foreign bodies comprised the majority of identified foreign bodies.^19^ In our study, the majority of foreign bodies were pieces of bone (chicken bone 42.9% and fishbone 2.5%) and meat boluses. The other foreign bodies included food boluses, fruit seeds, dentures, wire, coin, button, hair, stones were found in each patient (0.8%). In our study, most patients were in the age group of 50-74 years (38.7%), followed by 25-49 years (32.8%). The most common age group was the fifth decade of life in a previous study from Nepal and India.^23^

Different endoscopic methods and equipments were used depending upon the types of foreign bodies. Food bolus was removed either in bloc or in a piecemeal fashion with help of a retrieval basket or forceps. Endoscopic management was done in 109 patients. Among them, complete extraction of foreign bodies was achieved with the help of Dormia basket in 58 patients (84.1%), followed by various types of forceps or snare. Gentle pressure was applied with the tip of the endoscope to the esophageal food bolus, following air insufflation The most frequently used accessory devices were retrieval baskets (n = 69, 58 %), retrieval forceps, or polypectomy snares (n =50, 42 %). Other studies also showed retrieval net, rat tooth forceps as common retrieval systems used. It is suggested from animal studies and clinical practice that the retrieval net is appropriate for the removal of smooth objects (coins, disc batteries, or magnets).^27-8^

In our study, one denture without wire could be removed from the esophagus but failed to retrieve in 4 patients (80%). Open thoracotomy was required in one patient to remove such a foreign body. Those foreign bodies are likely to cause laceration and perforation because of impaction in the esophagus. Ill-fitting dentures may easily dislodge in the esophagus during chewing food or drinking. Three (75%) cases were referred to the otolaryngology department. A similar study from the ENT department of the same center showed meat and bone (68.33%) were the commonest foreign body in the esophagus, followed by dentures (15.5%). All dentures were removed under conscious sedation but one required open thoracotomy.^25^ We reported a 98.8% success rate of endoscopic removal of foreign bodies and a 1.25% failure rate whereas a study from China success rate was 94.1% and the failure rate was 5.9%. Most of the foreign bodies that fail endoscopic removal were dental prostheses, iron slices, or complex and ultra-long objects.^5^

In a study done by Yao CC, et al. endoscopic management was a safe and highly effective procedure in extracting foreign body ingestion and food bolus impaction. 93.5% of foreign bodies in the current study cohort were successfully extracted and 5 patients required surgical interventions.^29^ Similarly Wu WT, et al. also focused on the early intervention within 24 hours for minimizing the complications. A total of 172 patients (52.7%) were found to have ingested foreign bodies; 73.5% were removed smoothly, 10.3% were treated by push technique and 16.0% with failed retrieval received alternative treatments.^30^

The most common underlying pathology was esophageal stricture (38 cases, 53.5%). Nearly half of the patients (49.9%) developed complications. As the duration of impaction increased, the success rate by endoscopy decreased.^19^

## CONCLUSIONS

Most upper GI foreign bodies in adults are related to food bolus impaction with bone and meat. Therapeutic endoscopy by an experienced skilled endoscopist with suitable accessories is a reliable, safe, and effective method in managing foreign bodies from the upper gastrointestinal tract.
